# Inclusive Education without Paradigm Change: Explaining Thailand’s Misalignment with the Salamanca Vision

**DOI:** 10.12688/f1000research.188764.1

**Published:** 2026-08-19

**Authors:** Pongmanut Deeod

**Affiliations:** 1 Faculty of Humanities and Social Sciences, Khon Kaen University, Nai Mueang, Khon Kaen, 40002, Thailand

**Keywords:** Inclusive Education, Salamanca Statement, Policy Paradigm, Universal Design for Learning, Thai Education Policy

## Abstract

**Background:**

Thailand has formally committed to inclusive education for three decades, yet its schools continue to fall short of the transformative vision set out in the Salamanca Statement. Existing explanations attribute this gap to implementation failure insufficient resources, weak enforcement, or inadequate training. This article argues instead that the persistent gap reflects a paradigm misalignment between Thailand’s welfare-oriented policy architecture and the rights-based model of disability underpinning international inclusive education norms.

**Methods:**

The analysis integrates policy paradigm theory, the rights-based model of disability under the Convention on the Rights of Persons with Disabilities, and Universal Design for Learning as a combined analytical framework. Thai legal instruments enacted since 1999, together with the empirical literature on their implementation, are synthesised to trace the depth of policy change achieved and to identify the structural conditions that have constrained it.

**Results:**

Legislative reform since 1999 has produced first- and second-order change new instruments, budget allocations, and support services without disturbing the single-standard logic that continues to organise curriculum, assessment, and teacher practice. Four interlocking structural conditions sustain this misalignment: a school system built around the average learner, a charity-oriented framing of disability, a standardised assessment regime, and a teacher role confined to curriculum implementation rather than learning design. The analysis proposes a theoretical mechanism, termed the “moral trap,” describing how a culturally legitimised charity ethic absorbs rights-based reform and shields the existing paradigm from structural challenge even after formal legal transformation.

**Conclusions:**

Building on these findings, the article proposes a Universal Design for Learning-Based Paradigm Realignment Framework, together with a four-question diagnostic tool, as a politically feasible pathway toward third-order change across curriculum, assessment, and teacher professional development one that does not require dismantling Thailand’s centralised bureaucratic structure.

## 1. Introduction

In 1994, the Salamanca Statement established a foundational reorientation in global education policy: schools should restructure to accommodate all learners, not select which learners fit existing structures ( UNESCO, 1994). This principle was subsequently codified as a legally binding human rights obligation through Article 24 of the Convention on the Rights of Persons with Disabilities (CRPD), which obligates States Parties to ensure inclusive education systems at all levels ( United Nations, 2006). General Comment No. 4 further specified that inclusion requires systemic transformation of educational culture, policy, and practice not merely placement of disabled learners in mainstream settings and that individual support must not substitute for accessible system design ( Committee on the Rights of Persons with Disabilities, 2016; de Beco, 2014; UNESCO, 2017). Together, these instruments define inclusion as a structural obligation rather than a service arrangement.

Despite three decades of international norm diffusion, however, a persistent gap remains between inclusive education as a rights-based ideal and its realisation in practice. The literature identifies a fundamental conceptual ambiguity: the term “inclusion” encompasses practices ranging from physical placement of disabled students in mainstream classrooms, through specialist service provision within schools, to whole-system redesign for learner diversity ( Göransson & Nilholm, 2014; Hardy & Woodcock, 2015). This semantic breadth enables systems to characterise their practices as inclusive even when curriculum reduction, ability grouping, or activity separation for specific learner groups persists. As Haug (2017) argues, evaluating inclusive education requires examining alignment across policy principles, school structures, instructional practice, and learner experience not enrolment statistics alone. Physical co-location, in other words, does not constitute genuine inclusion when curriculum and assessment remain organised around assumptions of learner uniformity ( Ainscow et al., 2019).

This structural problem is reinforced by modern educational systems that continue to organise learning through standardised curricula, competitive assessment, and single-criterion performance standards. Such mechanisms define which learners are “normal” and which require remediation, encoding diversity as individual deficit rather than as a design challenge for the system ( Miles & Singal, 2010; Singal, 2008). When schools expand access without altering these underlying mechanisms, physical inclusion coexists with curricular and pedagogical exclusion a pattern documented across diverse national contexts ( Waitoller & Artiles, 2013). Addressing this pattern requires changes not only to policy instruments but to the foundational assumptions governing how schools, curricula, and assessment are designed.

The teacher literature reinforces this conclusion. Inclusive education success does not depend on disability-specific knowledge alone; it requires a reconceptualisation of the teacher’s role from curriculum implementer to learning designer ( Florian & Black-Hawkins, 2011). Inclusive pedagogy proposes that teachers design learning opportunities flexible enough for all learners from the outset, rather than planning a standard lesson and differentiating for individuals afterwards ( Sharma et al., 2008). Yet this role shift cannot be achieved through training alone; it requires changes to the institutional conditions curriculum structure, assessment design, and professional accountability frameworks that constrain what teachers can do ( Forlin & Chambers, 2011). Universal Design for Learning (UDL) operationalises this principle at the system level, proposing that learning goals be decoupled from access and demonstration modalities so that no single instructional format becomes an unnecessary barrier ( Rose & Meyer, 2002; CAST, 2024). UDL Guidelines version 3.0 extend this logic to address institutional bias and systems of exclusion, positioning UDL as an organisational design principle rather than a classroom technique one that must be connected to curriculum, assessment, and teacher development to avoid reduction to mere media supplementation ( Capp, 2017; Rao et al., 2014).

Thailand exemplifies the gap between rights adoption and systemic transformation. The National Education Act B.E. 2542 (1999) and the Education for Persons with Disabilities Act B.E. 2551 (2008) formally incorporated inclusive principles, guaranteeing access to mainstream schools and mandating individualised support. Yet empirical studies consistently report that these legal commitments have expanded enrolment without transforming learning conditions. Schools predominantly respond to learner diversity through add-on specialist services—special education teachers, teaching assistants, and individual education plans—within an otherwise unchanged curricular and assessment structure ( Bualar, 2016; Kantavong et al., 2012; Vorapanya & Dunlap, 2014). Evidence across diverse Thai contexts, from local-government primary schools to universities, shows that formal rights do not guarantee equitable access to learning: leadership culture, teacher attitudes, conceptual ambiguity about inclusion, and family involvement all constrain practice, yet increasing support provision has not resolved the underlying structural pattern ( Bualar, 2018, 2024; Kantavong, 2018; Klibthong & Agbenyega, 2022; Sukbunpant et al., 2013).

Collectively, these studies illuminate important implementation barriers but leave a deeper question unanswered: why do additional resources and policy instruments continue to be absorbed into individual remediation logic rather than driving systemic change? This recurrent pattern three decades of reform producing the same reported obstacles suggests that the problem lies not in what is missing from implementation, but in what has not changed at the paradigmatic level. Policies transposed from international norms are always interpreted through existing regulations, bureaucratic structures, and institutional routines ( Ball et al., 2011; Bromley & Powell, 2012). Rights-aligned language can therefore coexist with practice built on unchanged assumptions about standard learners, normal learning, and systemic responsibility ( Dolowitz & Marsh, 2000). What must be examined is not only which additional instruments the state has added, but whether the foundational paradigm governing school design, curriculum, and assessment has changed.

This article argues that Thailand’s inclusive education gap is better understood as a paradigm misalignment than as an implementation failure. Drawing on Hall’s (1993) policy paradigm theory, the CRPD rights-based model, and UDL, it analyses why legal and programmatic reforms have remained at the first and second orders of change adjusting instruments and settings without reaching the third order of paradigmatic transformation. Four interlocking structural conditions are identified as sustaining this misalignment: a school system designed around the average learner, a charity-oriented disability culture, a standardised assessment regime, and a teacher role defined as curriculum implementation rather than learning design. Building on these findings, the article proposes a UDL-Based Paradigm Realignment Framework as a practical mechanism for achieving third-order change within Thailand’s centralised educational system and offers a diagnostic tool applicable to any country pursuing Salamanca- or CRPD-based reform.

## 2. Research objectives

1 To analyse the paradigmatic causes of the gap between Thailand’s inclusive education policy and the Salamanca Statement.
2 To propose a UDL-Based Paradigm Realignment Framework for inclusive education policy in the Thai context.

## 3. Theoretical foundations

Explaining why Thailand’s inclusive education policy has failed to fulfil the intent of the Salamanca Statement requires theoretical frameworks capable of distinguishing between surface-level formal change and transformation of the foundational assumptions governing state action. This article synthesises three complementary frameworks that form an integrated analytical chain: policy paradigm theory diagnoses why change remains stuck at the ideational level; the rights-based disability model under the Convention on the Rights of Persons with Disabilities establishes the normative standard against which genuine inclusion should be measured; and Universal Design for Learning provides the practical design mechanism through which paradigmatic change can be operationalised in policy. Each framework performs a distinct function, yet together they constitute a single logical sequence for analysing and addressing Thailand’s paradigm misalignment.

### 3.1 Policy paradigm theory

Hall (1993) proposes that state policy change occurs at three levels of increasing depth. First-order change adjusts the settings of existing policy instruments adding budget allocations or personnel without altering goals or foundational assumptions. Second-order change modifies the instruments themselves, introducing new tools while leaving the overarching framework intact. Third-order change, the deepest level, alters the foundational paradigm: the framework of ideas that determines which problems are recognised as problems and which solutions are considered legitimate within the bureaucratic system. This framework is particularly valuable for the Thai case because it reveals that Thailand’s rapid legislative adoption of inclusive education rights from 1999 onwards constitutes first- and second-order change only. The state added legal instruments and support mechanisms without disturbing the single-standard logic that continues to organise the education system at its core. What this article terms “welfare inclusion” the provision of individual support services within an otherwise unchanged system is therefore not a failure of policy implementation but a predictable outcome of reforms that never reached the paradigmatic level ( Dolowitz & Marsh, 2000).

### 3.2 The CRPD and the rights-based model of disability

If the Salamanca Statement established the founding aspiration for inclusive education internationally, the Convention on the Rights of Persons with Disabilities (CRPD) ratified by Thailand in 2008 provides the legally binding normative standard that specifies more precisely what inclusion must mean. General Comment No. 4 on the right to inclusive education ( Committee on the Rights of Persons with Disabilities, 2016) makes clear that inclusion is not the provision of additional services for learners with special needs within an existing system, but the structural reform of educational culture, policy, and institutional practice as a whole. This position is grounded in the rights-based model of disability, which holds that barriers to participation arise not from individual impairment but from environments and social systems that have not been designed to accommodate human diversity from the outset ( Oliver, 2018; Shakespeare & Richardson, 2018). This model stands in direct opposition to the charity-oriented approach, which frames disability as a condition requiring compassionate remediation a framing this article identifies as deeply embedded in Thai educational culture. As Shaeffer (2019) argues, the transition from charity-oriented to rights-based models of disability is a necessary condition for educational justice at the international level. Employing the CRPD and the rights-based model as comparative benchmarks in this analysis is therefore not merely a sociological reference point; it establishes a legally verifiable standard against which the degree of Thailand’s deviation from its own ratified international obligations can be assessed ( de Beco, 2014; United Nations, 2006).

### 3.3 Universal design for learning

While the first two frameworks explain why the gap exists and establish the standard by which success should be measured, Universal Design for Learning (UDL) functions as the practical operational mechanism connecting rights-based principles to concrete policy and learning system design. Developed from universal design principles in architecture, UDL proposes that effective learning environments must be designed to accommodate learner diversity from inception, rather than being designed for the majority and subsequently adapted for those who do not conform to the standard. CAST (2024) articulates three core UDL principles: multiple means of representation, multiple means of action and expression, and multiple means of engagement. These principles align directly with the inclusive pedagogy framework of Florian and Black-Hawkins (2011), which holds that genuine inclusion occurs when teachers design core learning activities to be responsive to diversity from the outset, rather than planning a standard lesson and providing separate activities for specific learners afterwards. The updated UDL Guidelines version 3.0 further extend these principles to address institutional bias, identity, sense of belonging, and systems of exclusion, establishing UDL as an institutional design principle rather than merely a classroom technique ( Capp, 2017).

**Figure 1.**
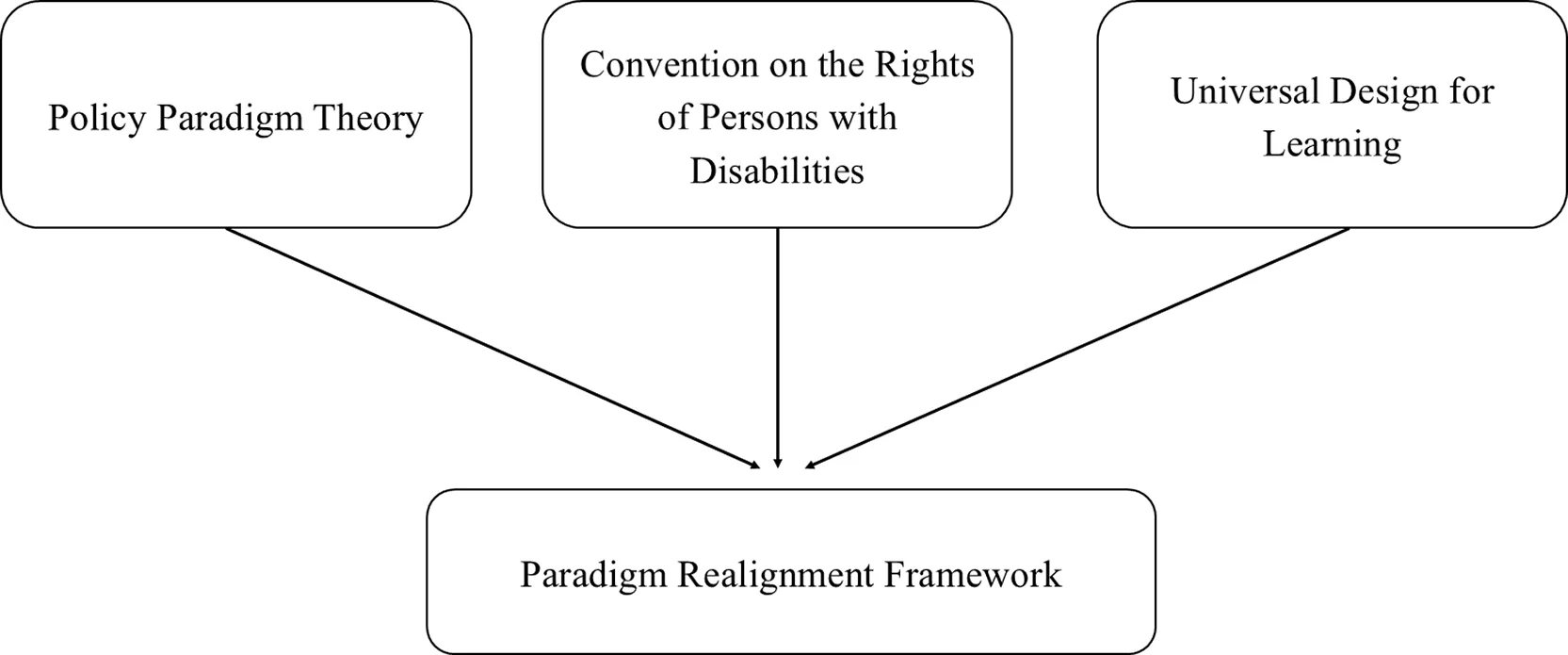
Three-Part Theoretical Framework for Analysing the Gap between Thailand’s Inclusive Education Policy and the Salamanca Statement.

The relevance of UDL to the Thai context lies in its political and administrative feasibility. UDL does not demand the dismantlement of the centralised curriculum framework or the wholesale restructuring of the bureaucratic state; it proposes changing the design logic operating within existing structures. This makes it a practically achievable pathway to paradigm-level change in ways that more radical calls for institutional transformation are not which is precisely why this article positions UDL as the central mechanism of the policy framework proposed in subsequent sections. When the three frameworks are integrated, a coherent analytical logic emerges. Policy paradigm theory establishes that Thailand’s inclusive education problem is paradigmatic rather than instrumental. The CRPD and rights-based disability model provide the legal and theoretical standard by which the Thai state’s interpretation of inclusion can be assessed against its own international commitments. UDL then offers the practical operational pathway through which paradigm change can be realised without dismantling the existing bureaucratic system. The three frameworks thus do not operate independently but constitute a single integrated lens for reading both the problem and the solution of inclusive education in Thailand, as illustrated in Figure 1.

## 4. Method and analytical strategy

This article employs conceptual analysis an approach suited to clarifying contested concepts, synthesising theoretical frameworks, and developing explanatory models ( Jaakkola, 2020). Conceptual analysis is appropriate here because the central question why paradigm-level change has not occurred in Thailand is primarily an interpretive and theoretical problem, requiring comparison of normative frameworks against documented policy patterns rather than new data collection. The approach differs from a systematic literature review in that its primary aim is framework development rather than evidence aggregation, and from a case study in that it does not rely on primary fieldwork.

### 4.1 Document corpus and selection

The analysis draws on two document streams. First, key Thai legal and policy texts governing inclusive education: the National Education Act B.E. 2542 (1999), the Education for Persons with Disabilities Act B.E. 2551 (2008), the Basic Education Core Curriculum B.E. 2551 (2008), and Ministry of Education guidelines on inclusive practice. These were selected because they constitute the primary instruments through which international norms have been formally adopted. Second, peer-reviewed empirical studies on inclusive education implementation in Thailand, published in Thai and English between 1999 and 2024. Studies were included if they (a) focused on Thai school or policy contexts, (b) reported primary data or systematic analysis, and (c) were published in indexed journals or comparable scholarly venues. International theoretical and normative literature was selected to represent foundational frameworks ( Hall, 1993; CRPD; UDL) and to contextualise Thai findings within global patterns of inclusive education research.

### 4.2 Analytical procedure

Analysis proceeded in three stages. In Stage 1, policy documents were coded against Hall’s three orders of change to determine whether reforms addressed instruments, settings, or the underlying paradigm—asking specifically whether each reform altered the foundational assumptions about learners and learning design. In Stage 2, empirical findings from the Thai literature were mapped onto four analytical domains curriculum design, disability framing, assessment, and teacher role to identify recurring patterns of paradigm-level continuity despite surface-level change. In Stage 3, findings were synthesised against CRPD standards and translated into framework components using UDL design principles, producing the Paradigm Realignment Framework presented in Section 7.

## 5. Findings

Analysis of legal documents, policy texts, and empirical literature within the analytical framework established in the methodology yields three principal findings: (1) Thailand has integrated inclusive education principles clearly at the legislative and policy levels, yet paradigmatic change within the education system has not occurred; (2) the gap between policy and practice reflects reforms that have remained primarily at the level of instrument adjustment and support measures rather than structural transformation of the learning system; and (3) synthesis of all evidence leads to the identification of four structural conditions that explain the misalignment between the intent of the Salamanca Statement and Thailand’s policy implementation.

### 5.1 Thailand has adopted inclusive education principles at the policy level without achieving paradigmatic change in the education system

Analysis of Thailand’s legal and policy documents reveals that Thailand was among the countries that responded to inclusive education relatively rapidly following the proclamation of the Salamanca Statement, progressively integrating educational rights principles into national legislation. Beginning with the National Education Act B.E. 2542 (1999), which affirmed education as a fundamental right of all citizens, Thailand subsequently enacted the Education for Persons with Disabilities Act B.E. 2551 (2008), guaranteeing the right of persons with disabilities to access education in general educational institutions and obligating the state to provide assistive facilities, technology, adapted media, and individualised support services (National Education Act B.E. 2542, 1999; Education for Persons with Disabilities Act B.E. 2551, 2008). Compared against the principles of the Salamanca Statement ( UNESCO, 1994) and the Convention on the Rights of Persons with Disabilities ( United Nations, 2006), Thailand’s legislative framework demonstrates alignment with international standards at the level of principle: it acknowledges that learner difference is not a barrier to educational entitlement and establishes state obligations to provide support systems ensuring equitable access for all learners. These principles are further consistent with General Comment No. 4 of the Committee on the Rights of Persons with Disabilities, which specifies that inclusive education is a right, not a welfare service or specialist provision for disabled persons ( Committee on the Rights of Persons with Disabilities, 2016).

However, comparative analysis of these policy documents against Hall’s (1993) policy paradigm framework reveals that the changes described reflect adaptation primarily at the level of law and policy instruments. The foundational assumptions of the education system continue to centre the standard learner in curriculum design, instructional organisation, and assessment. That is, while the state has extended rights and support services to learners with special educational needs, the core structure of schooling remains premised on the notion that learners should progress through a single curriculum, a single pedagogical approach, and a single set of assessment criteria. Learner difference continues to be managed through the addition of individualised services rather than through systemic redesign for flexibility and diversity. This finding is consistent with international literature proposing that many countries achieve “policy inclusion” substantially earlier than “structural inclusion” ( Göransson & Nilholm, 2014; Haug, 2010; Slee, 2011). Legal recognition of inclusive principles does not automatically transform how the education system defines learners, success, and difference. Where curriculum structure, measurement, and school administration continue to operate under a single-standard logic, the addition of rights or support services tends to be absorbed into the existing system rather than transforming it.

Analysed through Hall’s (1993) policy change framework, the first principal finding of this study is therefore that Thailand has clearly integrated inclusive education principles at the legislative and policy levels, but that these changes have not advanced to the paradigmatic level of the education system. Numerous instruments and support measures have been adjusted, yet no clear evidence emerges that the foundational paradigm governing school design, curriculum, and learning has shifted from “accommodating the standard learner” to “designing for learner diversity” as genuinely intended by the Salamanca Statement and the Convention on the Rights of Persons with Disabilities.

### 5.2 Empirical evidence from policy implementation

Following the analysis of legislation and policy, this study examined empirical research in both Thai and English examining inclusive education practice in Thailand, in order to consider whether adoption of inclusive principles at the policy level has led to transformation of the learning system in accordance with the intent of the Salamanca Statement. Synthesis of the evidence reveals a consistent pattern: despite differences in institutional context, studies uniformly reflect that changes have primarily involved adding support measures within the existing educational structure rather than modifying the structure of curriculum, instructional design, and assessment to accommodate learner diversity from the outset ( Kantavong et al., 2012; Vorapanya & Dunlap, 2014; Sukbunpant et al., 2013). Evidence across multiple studies indicates that the majority of schools continue to apply a standard national curriculum, uniform instructional approaches, and common assessment criteria to all learners, while responses to learner difference occur through the addition of support services specialist teachers, teaching assistants, individual education plans, or partial activity modification for specific students without altering the core structure of classroom learning ( Bualar, 2016; Office of the Basic Education Commission, 2008). This pattern reflects inclusion enacted through additional resources to help learners adapt to the system, rather than through systemic adaptation to learner diversity.

This finding is consistent with international scholarship proposing that expanding access to mainstream schools does not constitute genuine inclusive education when curriculum, pedagogy, and assessment remain premised on assumptions of learner uniformity ( Ainscow, 2005; Haug, 2017). Physical co-location does not equate to shared learning when specific learner groups continue to depend on individualised assistance to function within an unchanged system. Analysed through Hall’s (1993) policy paradigm framework, these findings indicate that Thailand’s inclusive education reforms have occurred primarily at the level of policy instrument adjustment and instrument settings adding support services, developing guidelines, and allocating additional resources without evidence of change at the level of the policy paradigm itself, meaning a fundamental shift in how schools, curricula, and learning processes are conceptualised to accommodate learner diversity as a normal condition of the system.

Measured against the rights standard established by the Convention on the Rights of Persons with Disabilities and General Comment No. 4 which specify that inclusive education must constitute structural reform of the education system rather than merely the provision of additional services to individual learners ( United Nations, 2006; Committee on the Rights of Persons with Disabilities, 2016) the analysis reveals that Thailand continues to face a gap between rights recognition at the policy level and structural transformation of learning provision. This gap reflects not merely resource constraints or implementation limitations, but a misalignment between the rights-based principles articulated in policy and the foundational assumptions that continue to govern the operation of the Thai education system.

This finding raises the central analytical question of the study: why has sustained reform over three decades been unable to change the foundational logic of the education system? Synthesis of evidence from policy documents, empirical research, and the three theoretical frameworks together leads to the finding that this phenomenon is not incidental, but arises from structural conditions embedded within the Thai education system, which can be identified as four distinct and mutually reinforcing conditions, presented in the following sections.

### 5.3 Four structural conditions

**5.3.1 A school system designed around the average learner**

The first structural condition explaining the misalignment between Thailand’s inclusive education policy and the intent of the Salamanca Statement is that the Thai education system continues to operate under a standardisation logic that organises the educational process around the average learner as the central reference point. Despite legal and policy recognition of the rights of diverse learners, the foundational structure of curriculum, instructional design, and assessment continues to require all learners to attain the same outcomes within the same timeframe, through the same learning methods, and against the same evaluative criteria. Documentary analysis reveals that the Basic Education Core Curriculum B.E. 2551 (2008) establishes common learning standards and performance indicators for learners at each educational level across the country, with the aim of producing nationally consistent, manageable educational quality ( Office of the Basic Education Commission, 2008). While this approach provides clarity in standard-setting, it simultaneously produces a learning design logic premised on the assumption that learners can acquire knowledge through a single pathway within comparable timeframes, with the result that learner difference is managed through post-hoc individualised assistance rather than through system design that accommodates diversity from inception.

This finding is consistent with research both in Thailand and internationally reporting that the majority of schools continue to apply a single curriculum, single instructional approach, and single assessment criteria to all learners, even when students with special educational needs are enrolled in the same classroom ( Bualar, 2016; Kantavong et al., 2012; Göransson & Nilholm, 2014). In practice, schools characteristically respond by adding specialist teachers, teaching assistants, or individual education plans while leaving the core instructional structure unchanged, with the result that learners with differences must adapt to the system rather than the system adapting to accommodate learner diversity. Analysed through Hall’s (1993) policy paradigm framework, this reflects reform confined to the addition of policy instruments and support measures without altering the foundational assumptions of the education system. The state has added resources and services for learners with special needs without displacing the average-learner paradigm that organises curriculum design, instruction, and assessment. Learner difference accordingly continues to be interpreted as a systemic exception rather than as a normal condition that the system should be designed to accommodate.

This finding also reflects a significant divergence between the Salamanca Statement’s intent and Thailand’s policy practice. The Salamanca Statement proposes that schools restructure their learning systems to accommodate all learners from the outset ( UNESCO, 1994), while Thailand’s operational approach continues to prioritise maintaining the standard school structure and providing supplementary services for learners unable to access learning within that structure. The result is that inclusion is enacted as “adding learners to the existing system” rather than “redesigning the system for diverse learners.” Standardisation-based bureaucracy is thus not merely an administrative characteristic of Thai education but a structural condition that shapes how learners, success, and learning are conceptualised across the entire system. As long as curriculum design, assessment, and school administration remain premised on the average learner, inclusive education will tend to be enacted as support service addition rather than genuine paradigm transformation.

**5.3.2 The framing of disability as a condition requiring assistance rather than as a rights-bearing status**

The second structural condition explaining the gap between Thailand’s inclusive education policy and the intent of the Salamanca Statement arises not primarily from legal or resource constraints, but from the conceptual framework used to interpret disability and learner difference, which continues to reflect a charity-oriented approach rather than recognition of all learners as rights holders entitled to an education system designed to accommodate difference equitably. Analysis of Thai legislation and policy reveals that although multiple statutes formally affirm the right of persons with disabilities to access education and require the state to provide assistive facilities, adaptive media, technology, and individualised support, the operational mechanisms of policy implementation remain oriented primarily toward providing additional services to specific learner groups rather than requiring schools to redesign their learning provision for all learners. In practice, the right to inclusive education is interpreted primarily through “receipt of services” rather than through systemic redesign for accessibility by design (Education for Persons with Disabilities Act B.E. 2551, 2008; Office of the Basic Education Commission, 2008).

This finding is consistent with Thai research reporting that many schools continue to regard learners with special educational needs as a “specific target group” requiring special case management, resulting in inclusion enacted primarily through individualised service provision—specialist teachers, teaching assistants, or individual education plans—while whole-class learning design remains unchanged ( Kantavong et al., 2012; Vorapanya & Dunlap, 2014; Bualar, 2016). Such practice reflects an interpretation of difference as a characteristic of the individual learner requiring compensation rather than as an outcome of limitations in the education system itself. Measured against the framework of the Convention on the Rights of Persons with Disabilities and General Comment No. 4, inclusive education rests on a substantially different principle: disability is not a characteristic of an individual but arises from the interaction between individual difference and physical, social, and institutional barriers ( United Nations, 2006; Committee on the Rights of Persons with Disabilities, 2016). The state’s obligation is therefore not merely to provide additional assistance but to eliminate barriers that the education system itself produces and to design learning environments in which all learners can participate equitably.

These findings are consistent with the arguments of Oliver (2018) and Shakespeare & Richardson (2018), who explain that the transition from the medical model to the social and rights-based model of disability does not mean rejecting individualised support, but shifting the emphasis from correcting individual deficits to transforming the social and institutional structures that generate exclusion. Where the education system continues to rely on individualised service provision as its primary mechanism of inclusive education, without transforming curriculum, pedagogy, and assessment, disability remains framed as “the learner’s problem” rather than as a systemic responsibility. Analysed through Hall’s (1993) framework, Thailand’s expansion of rights and support services reflects instrument-level policy change, while the foundational paradigm governing disability continues to frame difference management through compensatory measures rather than systemic redesign. Inclusive education thus continues to be enacted as “assistance policy” rather than “rights policy,” despite formal legal recognition of the right to inclusion.

**5.3.3 A standardised assessment regime**

The third structural condition preventing Thailand’s inclusive education from fulfilling the intent of the Salamanca Statement is a measurement and assessment system that continues to rely on single-standard assessment as the primary mechanism for determining learner success. Despite policy recognition of the rights of diverse learners, the assessment process continues to assume that all learners should demonstrate learning through the same methods and against the same criteria, with the result that learner difference is interpreted as an individual limitation rather than as a reflection of constraints in the learning system itself. Documentary analysis of Thailand’s policy and curriculum reveals that the education system continues to prioritise nationally determined learning standards, performance indicators, and achievement outcomes, using assessment as a central mechanism for regulating school quality and learner development ( Office of the Basic Education Commission, 2008; Office of the Basic Education Commission, 2008). While provisions exist for teachers to modify assessment methods for specific learner groups, in practice the majority of assessment remains anchored to single-standard outcomes as the principal criterion of educational success, such that assessment modification is characteristically an exception rather than a foundational principle of learning design.

Evidence from both Thai and international research consistently supports this finding: even where schools admit learners with special educational needs to general classrooms, when assessment continues to apply the same criteria to all learners, teachers tend to adapt learners to fit the assessment system rather than adapting the assessment system to reflect the different learning approaches of individual students ( Waitoller & Artiles, 2013). The outcome is that a substantial number of learners can “access school” without being able to access a fair opportunity to demonstrate their capabilities within the prevailing assessment system. Under the rights framework of the Convention on the Rights of Persons with Disabilities, assessment is not merely an academic measurement tool but a core component of the right to equitable education, since inflexible assessment methods can become barriers to participation no less significant than physical environmental or access constraints ( United Nations, 2006; Committee on the Rights of Persons with Disabilities, 2016). Inclusive education therefore does not simply mean enabling learners to sit the same examination; it means designing an assessment system capable of reflecting learning outcomes through multiple methods while maintaining academic standards.

This finding is directly consistent with UDL principles, which propose separating “learning goals” from “methods of demonstrating learning” learners can achieve the same outcomes through different means without requiring a single assessment format ( Rose & Meyer, 2002; CAST, 2024; Rao et al., 2014). Assessment should function as a mechanism for reflecting learner progress rather than as a mechanism for sorting learners into those who succeed and those who fail against a single standard. Analysed through Hall’s (1993) framework, although Thailand has progressively developed guidelines, frameworks, and support measures for assessment of learners with special educational needs, these changes remain at the level of instrument adjustment rather than paradigmatic change in the conceptual role of assessment within the education system. The system continues to treat assessment as a mechanism for screening and comparing learner achievement rather than as a tool for supporting the learning of diverse learners. Single-standard assessment is thus not merely an academic tool but a structural condition that influences the interpretation of learner capability, success, and value across the entire system.

**5.3.4 A teacher role defined as curriculum implementation rather than learning design for diverse learners**

The fourth structural condition preventing Thailand’s inclusive education from fulfilling the intent of the Salamanca Statement is the positioning of the teacher role within an educational administration structure that emphasises compliance with the national curriculum and central standards rather than empowering teachers as learning designers capable of responding flexibly to learner diversity. Although policy aspires to inclusive teaching practice, the professional development system, quality assurance mechanisms, and educational evaluation frameworks continue to weight competence in implementing the national curriculum and centrally determined indicators as the primary measure of teacher effectiveness. Analysis of the Thai inclusive education literature reveals that the majority of policy proposals focus on increasing teacher knowledge about disability, providing special education training, or adding specialist support personnel in schools ( Klibthong & Agbenyega, 2022). While such measures are necessary for teacher capacity development, they continue to rest on the assumption that the problem of inclusion lies in “teacher limitations” rather than examining how the structure of the education system constrains teachers’ capacity for flexible learning design. Teacher development thus tends to focus on increasing skills for supporting individual learners rather than developing the capacity to design classrooms that accommodate all learners from the outset.

These findings are consistent with international scholarship proposing that inclusive education success depends not on increasing disability-category knowledge alone, but on transforming the teacher role from curriculum transmitter to designer of learning environments that enable all learners to participate ( Florian & Black-Hawkins, 2011). Under inclusive pedagogy, teachers do not plan separate activities for specific learner groups; they begin from designing learning activities sufficiently flexible for the full range of learners within the same classroom. Analysed through UDL, the teacher role is not limited to case-by-case instructional modification but encompasses designing goals, learning processes, instructional materials, and assessment to accommodate different learning approaches from inception ( Rose & Meyer, 2002). This reorients the central question from “which learners require additional assistance?” to “how should the learning system be designed so that all learners can learn together?” Learner difference is thus not an exception for teachers to correct afterwards but a foundational condition of learning design.

Taken together, these four conditions a school system designed around the average learner, the framing of disability as a condition requiring assistance, a standardised assessment regime, and a teacher role defined as system implementation are not independent phenomena but constitute components of a single paradigm that continues to govern how learners, success, and educational provision are conceptualised in Thailand. Under this paradigm, inclusion is enacted as the addition of support measures for learners who differ rather than as structural redesign of the education system to accommodate diversity from the outset. The four principal findings therefore lead to the central conclusion of this study: the gap between Thailand’s inclusive education policy and the intent of the Salamanca Statement is not primarily caused by resource deficits or policy implementation failures, but by a paradigm misalignment between the rights-based principles affirmed in legislation and the foundational assumptions that continue to govern the design of the education system. This conclusion provides the essential basis for the development of the UDL-Based Paradigm Realignment Framework presented in the following section.

### 5.4 Thailand’s inclusive education gap arises from policy paradigm misalignment

Synthesising evidence from legal documents, policy texts, empirical research, and the three theoretical frameworks policy paradigm theory ( Hall, 1993), the rights-based disability model under the Convention on the Rights of Persons with Disabilities and Universal Design for Learning this study arrives at a central synthetic finding ( United Nations, 2006; Committee on the Rights of Persons with Disabilities, 2016) the gap between Thailand’s inclusive education policy and the intent of the Salamanca Statement is not primarily a failure of policy implementation, but an instance of policy paradigm misalignment between the rights-based principles legally affirmed and the foundational assumptions that continue to govern educational system design ( Rose & Meyer, 2002). Thailand has undertaken inclusive education reform across multiple dimensions enacting legislation, developing policy, allocating budgets, and building support systems for learners with special educational needs. However, these changes have occurred primarily at the levels of policy instruments and instrument settings rather than at the level of the foundational paradigm that determines how learning, capability, and learner success are defined. Despite policy affirmation of the right to inclusive education, curriculum design, assessment systems, teacher development, and school administration continue to rest on assumptions of the “standard learner,” such that the majority of measures are used to help learners adapt to the existing system rather than to redesign the system to accommodate diverse learners.

In Hall’s (1993) terms, this reflects first- and second-order policy change: the state can add legislation, measures, and support services without necessarily changing the foundational assumptions of the education system, producing alignment at the level of policy language while structural operational misalignment persists. The success of inclusive education should therefore not be assessed merely by the number of learners entering mainstream schools or the number of support measures the state provides, but by whether the education system has changed how it conceptualises learning design for learner diversity. This also explains why research continues to report the same obstacles repeatedly teacher unpreparedness, resource shortfalls, curriculum inflexibility, and assessment constraints despite three decades of policy implementation and development programmes. These measures have addressed problems at the operational level while the foundational assumptions governing educational system design have remained unchanged. Additional resources can reduce certain barriers but cannot sustainably transform the foundational logic of the system.

This study therefore proposes that the policy-practice gap in Thai inclusive education arises not solely from implementation constraints but from the paradigm misalignment governing educational system design. The synthetic findings are summarised in Table 1, which illustrates the relationship between structural conditions, the paradigm currently governing educational provision, and the direction of transformation required to achieve inclusive education in accordance with the intent of the Salamanca Statement.

**Table 1.** Summary of Analytical Findings.

Structural condition	Current paradigm	Salamanca principle	Required transformation
Average learner	Standardization	Learner diversity	Flexible curriculum
Charity approach	Welfare	Rights	Rights-based support
Standard assessment	One standard	Multiple pathways	Flexible assessment
Teacher role	Implementer	Learning designer	UDL pedagogy
Overall system logic	Remedial support	Structural design for diversity	Inclusive by design

The direction of transformation required to achieve inclusive education in accordance with the intent of the Salamanca Statement. The final row synthesises the four structural conditions into an overarching system logic, providing the conceptual foundation for the fourth dimension of the UDL-Based Paradigm Realignment Framework developed in Section 7.

## 6. Discussion

The findings of this article support, extend, and challenge existing scholarship on inclusive education. First, they support the established conclusion that the formal adoption of inclusive education principles does not automatically produce inclusive practice ( Göransson & Nilholm, 2014; Haug, 2017; Slee, 2011). Consistent with previous studies, the Thai case demonstrates that legislative reform and policy commitments can coexist with limited transformation in curriculum, assessment, and classroom practice. However, the findings also extend this literature by offering a more explicit theoretical explanation for why implementation gaps persist despite sustained policy reform. Rather than viewing repeated implementation barriers solely as operational shortcomings, this study suggests that they are reproduced through an unchanged policy paradigm. Drawing on Hall’s (1993) framework, the analysis indicates that paradigm continuity is sustained by four interlocking structural conditions that reinforce one another institutionally, allowing additional resources and policy instruments to be absorbed into the existing system rather than destabilising its underlying assumptions.

The article also challenges accounts that explain Thailand’s inclusive education gap primarily through implementation barriers, such as insufficient resources, inadequate teacher preparation, or administrative fragmentation. These factors remain important and should not be dismissed; however, the findings suggest that they are better understood as manifestations of a deeper paradigmatic problem rather than as its primary cause. This interpretation is consistent with the policy paradigm perspective, which argues that enduring policy outcomes are shaped not only by the effectiveness of implementation but also by the underlying ideas that define legitimate policy goals, instruments, and problem definitions. Moreover, policy transfer research suggests that reforms borrowed from international frameworks are frequently reinterpreted through existing institutional arrangements and administrative traditions rather than replacing them outright ( Dolowitz & Marsh, 2000; Ball et al., 2011). As long as the design logic of schools, curricula, and assessment continues to assume an average learner and positions diversity as deviation from the norm, additional investments are likely to be channelled into compensatory support rather than systemic redesign, a pattern that reflects deficit-oriented understandings of difference in inclusive education policy ( Hardy & Woodcock, 2015; Graham & Slee, 2008). This interpretation shifts the analytical focus from improving the implementation of existing arrangements to examining the institutional assumptions that shape how reforms are interpreted and enacted ( Bromley & Powell, 2012). In this sense, the critical unit of change is not solely the individual teacher or school, but the design logic embedded within the education system itself.

Finally, the concept of the moral trap introduced in this article represents a theoretical extension beyond Hall’s (1993) original formulation of policy paradigms. While Hall explains how policy paradigms become stabilised through institutional learning and established policy ideas, his framework does not explicitly address the role of culturally legitimised moral values in sustaining existing paradigms ( Hall, 1993). The Thai case suggests that where care-giving and charitable assistance are widely regarded as socially desirable responses to disability, welfare-oriented practices may reduce the perceived need for rights-based structural reform, even when legal obligations formally require such transformation ( Oliver, 2018; Shakespeare & Richardson, 2018). This proposition should be regarded as a theoretical hypothesis that warrants comparative empirical examination across societies with different cultural understandings of disability and care. More broadly, the findings reinforce the argument that rights-based policy language alone is insufficient to achieve paradigm change ( UNESCO, 2017). The CRPD defines the normative obligations of inclusive education, while UDL provides practical design principles for translating those obligations into curriculum, assessment, and teaching ( United Nations, 2006; Committee on the Rights of Persons with Disabilities, 2016; CAST, 2024). The Thai case demonstrates that both rights-based commitments and UDL principles can be formally incorporated into policy without altering the underlying assumption that education is organised around the average learner ( Ball et al., 2011). Closing this gap therefore requires translating rights-based commitments into concrete design requirements for curriculum, assessment, and teacher professional development, rather than relying primarily on the expansion of individual support services ( Florian & Linklater, 2010).

## 7. The UDL-Based paradigm realignment framework

**Figure 2.**
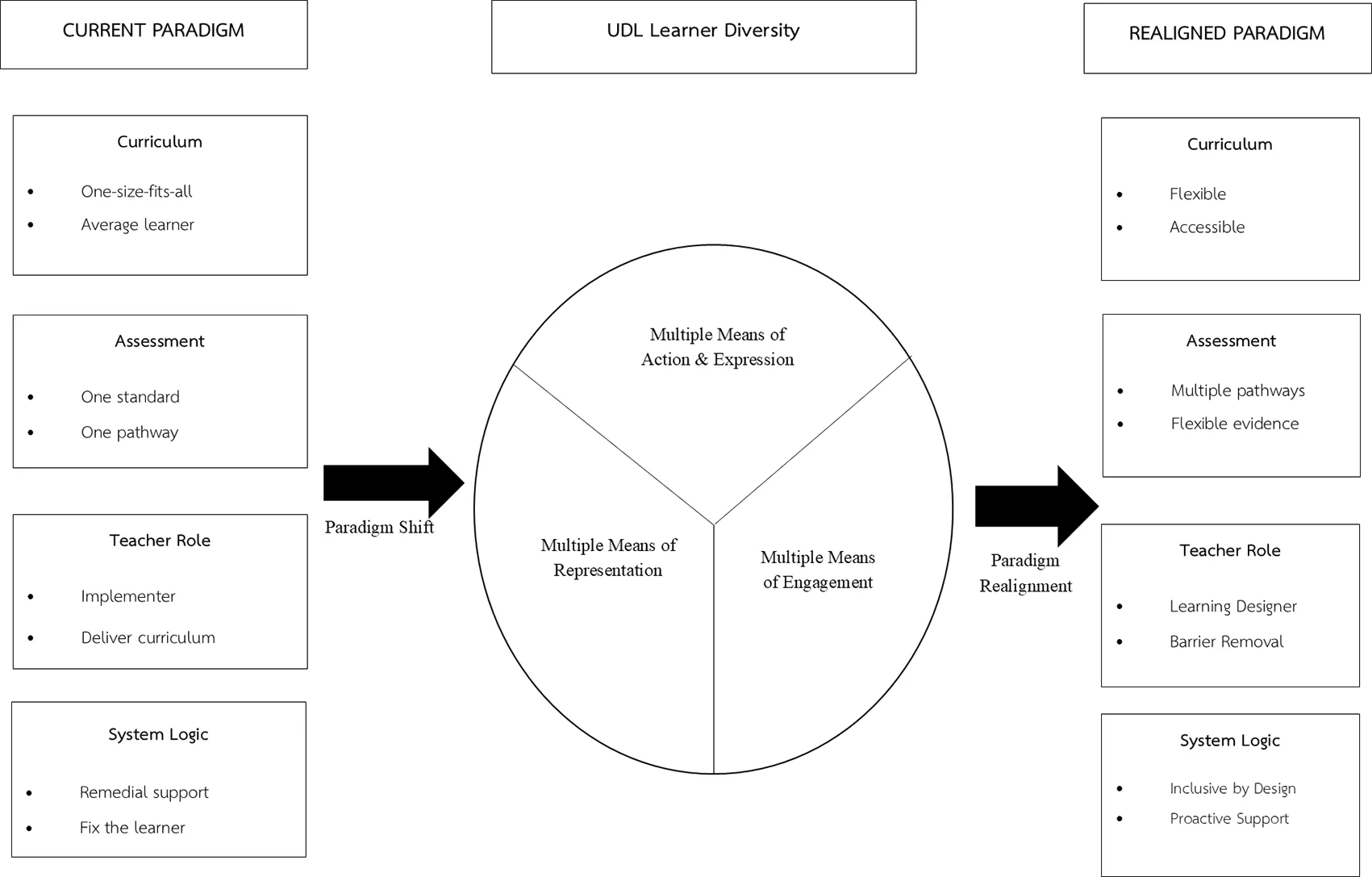
UDL-Based Paradigm Realignment Framework.

The UDL-Based Paradigm Realignment Framework proposed here is designed to operationalise the shift from first- and second-order reform to third-order paradigm change within Thailand’s centralised educational system (see Figure 2). It does not require wholesale restructuring of the bureaucratic state; rather, it proposes changing the design logic within existing structures across four policy dimensions.

Dimension 1: Curriculum. The framework proposes that the Basic Education Core Curriculum shift from specifying uniform pathways to specifying learning goals while permitting flexible routes to those goals. This is a UDL-aligned reconceptualisation of what standards specify: what students should know and be able to do, not how they must learn or demonstrate learning. This shift is achievable within the existing national curriculum framework through revision of implementation guidelines rather than wholesale curriculum redesign, making it politically and administratively feasible.

Dimension 2: Assessment. The framework proposes that national assessment policy mandate flexible evidence of learning multiple demonstration formats for the same learning objective as a default feature of the assessment system rather than an exception for individual students. This reframes assessment from a sorting mechanism to a learning-support tool, consistent with the CRPD’s conception of assessment as a dimension of the right to inclusive education. Flexible assessment by design does not lower academic standards; it separates the standard (what students must demonstrate) from the pathway (how they demonstrate it).

Dimension 3: Teacher Professional Development. The framework proposes that pre-service and in-service teacher education reorient from disability-specific knowledge transmission to inclusive learning design competence. Specifically, teachers should be trained and supported to design instructional units that anticipate learner variability from inception, not to modify standard lesson plans for identified students after the fact. This shift requires changes to both teacher education curricula and the accountability frameworks under which teachers work—teachers must have institutional authority to vary instruction without incurring professional risk.

Dimension 4: System Logic. The first three dimensions each redesign a specific policy instrument; this fourth dimension names the underlying operating principle that must change for the other three to hold together as paradigm-level reform rather than three isolated instrument adjustments. Under the current paradigm, learner difference is managed through remedial support extended after the standard system has already been designed and deliveredthe system is fixed, and the learner is the object of correction. The realigned system logic inverts this sequence: inclusive design is built into curriculum, assessment, and teaching from inception, and support becomes proactive rather than remedial anticipating variability rather than reacting to it once a learner has already failed to fit the standard pathway. This dimension is therefore not a discrete policy lever in the way the first three are; it is the integrating principle that determines whether curriculum, assessment, and teacher development reforms are coordinated expressions of a single redesigned logic or merely parallel accommodations layered onto an unchanged system.

The framework also incorporates a four-question diagnostic tool enabling policymakers to assess whether a proposed inclusive education reform operates at the paradigm level or merely adjusts instruments: (1) Does this reform change the design logic of the curriculum, or does it add support services within an unchanged curricular structure? (2) Does this reform reconceptualise assessment as flexible by default, or does it add accommodation exceptions? (3) Does this reform reposition teachers as learning designers with institutional authority to vary instruction, or does it train teachers to manage diversity within a fixed instructional frame? (4) Does this reform build inclusive design into the system from the outset, or does it retain a standard system and add remedial support after the fact? A reform that answers “adds” or “accommodates” to all four questions is operating at the first or second order and is unlikely to reduce the paradigm gap. A reform that answers “changes” or “reconceptualises” is operating at the third order and is more likely to generate durable inclusive practice. This diagnostic framework is applicable beyond Thailand: any country pursuing Salamanca- or CRPD-based reform can use it to assess the depth of proposed change before declaring policy success.

## 8. Conclusion

This article has argued that Thailand’s inclusive education gap is best understood as a paradigm misalignment rather than an implementation deficit. Despite three decades of legislative and programmatic reform, the foundational logic of Thai schooling that schools serve the average learner and accommodate deviations through remedial support has not been displaced. Four interlocking structural conditions sustain this paradigm: a standardisation-oriented curriculum, a welfare-oriented disability culture, a uniform assessment regime, and a teacher role that prioritises curriculum implementation over learning design. The UDL-Based Paradigm Realignment Framework proposed here offers a practical pathway to third-order change that does not require systemic dismantlement of centralised educational structures. By redesigning the logic of curriculum specification, assessment design, and teacher professional development, Thailand can create the conditions under which learner diversity is anticipated and designed for rather than accommodated after the fact.

The theoretical contributions of this article the concept of paradigm misalignment as a distinct category of inclusive education failure, and the moral trap hypothesis as a mechanism of paradigm stabilisation in charity-oriented cultural contexts open directions for comparative research. Future studies should test the diagnostic framework empirically across diverse national contexts, examine the political conditions under which third-order change becomes possible, and document the experiences of disabled learners, families, and teachers as primary stakeholders in paradigm transformation. Until the design logic of schools changes, legal rights to inclusive education will remain declarations rather than lived realities.

## Data Availability

No new primary data were generated in this study. The analysis is based on publicly available Thai legal and policy instruments, including the National Education Act B.E. 2542 (1999), the Education for Persons with Disabilities Act, and the Basic Education Core Curriculum B.E. 2551 (2008), together with previously published empirical literature. All source materials are cited in full within the reference list and are freely accessible through official government sources (e.g., the Royal Thai Government Gazette, Office of the Basic Education Commission) or through standard academic databases, enabling verification and reuse by other researchers. No individual, sensitive, or personal data were collected at any stage of this research, and the study did not involve human participants, interventions, or clinical data. Consequently, ethical approval or review by an Institutional Review Board (IRB) or equivalent ethics committee was not required. All procedures were conducted in accordance with international standards for research integrity and responsible data management.
