# Supplementary material for: Inclusive Education without Paradigm Change: Explaining Thailand’s Misalignment with the Salamanca Vision
Source: F1000Res. 2026 Sep 18;15:1395. Originally published 2026 Aug 19. [Version 2] doi: 10.12688/f1000research.188764.2 (PMC13579026; doi:10.12688/f1000research.188764.2)
Supplement: Peer review history

# Reviewer report: Reviewer response for version 2

**DOI:** 10.5256/f1000research.210707.r522397

**Published:** 2026-10-05

**Authors:** Siti Masyithoh

**Affiliations:** 1 Syarif Hidayatullah State Islamic University Jakarta, South Tangerang, Banten, Indonesia

**Recommendation: Approved with Reservations / Minor Revision**

 The revised manuscript has substantially addressed the major concerns raised in the previous review. The authors have strengthened the methodological transparency, clarified the evidentiary basis for the four structural conditions, repositioned the “moral trap” as an explicitly theoretical and testable proposition, clarified the conceptual status of the Paradigm Realignment Framework, incorporated counterarguments concerning standardisation, updated the literature, and added an appropriate limitations and future research section.

 The remaining issues are minor and primarily concern methodological completeness and proportionality of presentation. In particular, the manuscript would benefit from explicitly reporting the final corpus size and exclusion criteria, further reducing some repetition, and slightly moderating the conclusion so that the strength of the claims remains fully proportionate to the documentary and conceptual nature of the study.

**Overall decision: Approved with Reservations / Minor Revision.**

Is the work clearly and accurately presented and does it cite the current literature?

Partly

If applicable, is the statistical analysis and its interpretation appropriate?

Not applicable

Are all the source data underlying the results available to ensure full reproducibility?

Partly

Is the study design appropriate and is the work technically sound?

Partly

Are the conclusions drawn adequately supported by the results?

Partly

Are sufficient details of methods and analysis provided to allow replication by others?

No

Reviewer Expertise:

Character and Inclusive Education

I confirm that I have read this submission and believe that I have an appropriate level of expertise to confirm that it is of an acceptable scientific standard, however I have significant reservations, as outlined above.

# Reviewer report: Reviewer response for version 1

**DOI:** 10.5256/f1000research.208335.r513071

**Published:** 2026-09-15

**Authors:** Jonathan Chitiyo

**Affiliations:** 1 University of Pittsburgh Bradford, Bradford, Pennsylvania, USA

1) There is need to incoprate recent literature on inclusive education. I suggest the authors to consider the following articles: a) Bakari, A. S., & Juma, S. (2025). Barriers to Inclusive Education for Learners with Moderate Intellectual Impairment: Multiple Stakeholder Perspectives from Zanzibar Primary Schools. *International Journal of Whole Schooling*, *21*(2), 26-37.

 2) Authors need to provide some legal frameworks that guide the implementation of inclusive education in Thailand. Refer to the following: a) https://education-profiles.org/eastern-and-south-eastern-asia/thailand/~inclusion

 3) Thailand was once regarded as the model of inclusive education. This discussion needs to be intergrated in the literature review or discussion section.

Is the work clearly and accurately presented and does it cite the current literature?

Partly

If applicable, is the statistical analysis and its interpretation appropriate?

Not applicable

Are all the source data underlying the results available to ensure full reproducibility?

No source data required

Is the study design appropriate and is the work technically sound?

Yes

Are the conclusions drawn adequately supported by the results?

Partly

Are sufficient details of methods and analysis provided to allow replication by others?

Yes

Reviewer Expertise:

Special Educatio

I confirm that I have read this submission and believe that I have an appropriate level of expertise to confirm that it is of an acceptable scientific standard.

# Reviewer report: Reviewer response for version 1

**DOI:** 10.5256/f1000research.208335.r513067

**Published:** 2026-09-08

**Authors:** Madiha Khadim

**Affiliations:** 1 Govt. College Women University Faisalabad, Punjab, Pakistan

This is a conceptually ambitious and well-organised piece of policy-paradigm scholarship. Its central contribution — reframing Thailand's inclusive-education gap as *paradigm misalignment* rather than *implementation failure*, and proposing the "moral trap" as a candidate mechanism — is genuinely interesting and worth publishing in some form. The three-framework synthesis (Hall, CRPD/rights-based model, UDL) is coherently constructed and gives the paper real analytical structure, and the proposed Paradigm Realignment Framework with its four-question diagnostic tool has practical value as a policy-evaluation heuristic.

 The main issues a revision should address are:

- Methodological transparency: operationalise the document-coding scheme, report the literature search strategy and corpus size, and state  explicitly whether the four structural conditions were derived inductively or set a priori.
- Evidentiary weight for the moral-trap hypothesis ****either bring in supporting evidence (even secondary literature on Buddhist merit-making and disability stigma) or reframe it more cautiously throughout, not just in the closing paragraph.
- Reduce reliance on a small recurring set of Thai sources, and engage with disconfirming or mixed evidence to make the "pervasive stasis" claim more defensible.
- Explain why a reform proposed within the same bureaucratic-cultural structure would not be subject to the same absorption dynamic the paper diagnoses.
- Ensure the summary table 1 is complete in the submitted version.

**Recommendation:** *Accept with minor-to-moderate revisions* (revise methodology transparency, temper generalisability claims, and strengthen the empirical/literature basis for the moral-trap mechanism), contingent on these points being addressed. The theoretical contribution is sound and worth publishing; the manuscript primarily needs more rigor in showing its work rather than in the underlying argument itself.

Is the work clearly and accurately presented and does it cite the current literature?

Partly

If applicable, is the statistical analysis and its interpretation appropriate?

Yes

Are all the source data underlying the results available to ensure full reproducibility?

Yes

Is the study design appropriate and is the work technically sound?

Yes

Are the conclusions drawn adequately supported by the results?

Partly

Are sufficient details of methods and analysis provided to allow replication by others?

Partly

Reviewer Expertise:

My research area is Teacher Education. I went through this manuscript and found it worth for publishing.

I confirm that I have read this submission and believe that I have an appropriate level of expertise to confirm that it is of an acceptable scientific standard, however I have significant reservations, as outlined above.

# Response

**Published:** 2026-09-08

**Authors:** Pongmanut Deeod

Thank you for the constructive feedback. In the revision, we will: operationalize the coding scheme and report the search strategy and corpus size, clarifying whether the four structural conditions were derived inductively or a priori; strengthen and consistently temper the moral-trap hypothesis, drawing on secondary literature where possible; engage more with disconfirming evidence; address why a reform within the same bureaucratic-cultural structure would avoid the same absorption dynamic; and ensure Table 1 is complete. A revised version will be submitted shortly.

# Reviewer report: Reviewer response for version 1

**DOI:** 10.5256/f1000research.208335.r513069

**Published:** 2026-08-24

**Authors:** Siti Masyithoh

**Affiliations:** 1 Syarif Hidayatullah State Islamic University Jakarta, South Tangerang, Banten, Indonesia

Overall, the article presents a strong research question, interesting conceptual contributions, and a potentially useful framework, but still requires strengthening in its analytical methodology, evidence, claim accuracy, conceptual validation, and topicality of references. My recommendation is Approved with Reservations / Major Revision. Revisions should primarily focus on consistency between the evidence, analysis, claims, and contributions, so that the article not only offers a compelling conceptual argument but also has a strong scientific basis to support its conclusions.

 Therefore, I recommend the author improve the following points:

 1. Clarify the novelty of the article. Emphasize that the primary contribution is not the use of Hall, CRPD, or UDL, but rather the concept of paradigm misalignment, the moral trap proposition, and the proposed paradigm alignment framework.

 2. Strengthen the conceptual analysis methodology. Transparently explain the databases/search sources, search strategy, search period, number of documents and articles analyzed, inclusion and exclusion criteria, and the selection and coding process.

 3. Show an evidence trail. Systematically explain how the four structural conditions—average learner, welfare/charity framing, standardized assessment, and the role of teachers as implementers—are derived from policy documents and empirical research, not just from the author's interpretation.

 4. Avoid the dichotomy of "paradigm misalignment" versus "implementation failure." It is more accurate to state that implementation factors do not sufficiently explain gaps and that paradigm misalignment can be a structural condition that maintains implementation problems.

 5. Soften claims about a "charity-oriented culture." Available evidence supports the presence of welfare/charity-oriented elements or practices in certain policies and institutions, rather than the conclusion that Thailand's entire disability culture is charity-oriented.

 6. Position the moral trap as a theoretical proposition. This concept is interesting, but it has not been empirically tested. Do not present it as a proven mechanism. The article itself states the need for comparative empirical testing.

 7. Strengthen the connection between UDL and paradigm shift. Explain why UDL can be used at the policy and systems levels, not just as a classroom learning approach. If empirical evidence is lacking, state UDL as the proposed design mechanism.

 8. Affirm the Framework's status as a proposed/conceptual framework. The Framework and the four diagnostic questions have not been validated. Explain how they could be tested or validated in future research.

 9. Reduce the claim of "politically feasible." The article has not conducted a political or policy feasibility analysis. Use more careful terms such as "potentially compatible with the existing administrative structure."

 10. Strengthen the Discussion section. Don't simply state that the findings are "consistent with previous studies"; explicitly explain what is different, new, or more in-depth than previous research.

 11. Add counterarguments. Discuss the possibility that curriculum and assessment standardization also has positive functions, such as maintaining consistent standards and equity, before explaining why flexibility remains necessary in inclusive education.

 12. Update references. Add the most recent literature, especially from 2020–2026, on inclusive education in Thailand, UDL, disability rights, policy implementation, and policy paradigm change.

 13. Conduct a citation audit. Ensure that each citation fully supports the claim cited. Several references need to be re-examined for substantive consistency with the statements in the article.

 14. Clarify definitions of key terms. Conceptually differentiate between welfare, charity, assistance, accommodation, remediation, and rights-based inclusion to avoid overlapping use.

 15. Reduce repetition. The idea that Thailand added services without changing the system's structure appears repeatedly in the results, discussion, and conclusion sections. Some of this space should be allocated to strengthening the evidence and critical analysis.

 16. Soften overly absolute language. Terms such as "not implementation failure," "uniformly," "charity-oriented culture," and "will remain a declaration" should be replaced with formulations more in line with the nature of conceptual research.

 17. Strengthen conclusions to ensure they are commensurate with the method. Conclusions should clearly distinguish between findings from document/literature analysis, theoretical interpretations, and frameworks that are still propositional.

 18. Explicitly address the limitations of the study. For example, there is no primary data, no direct perspectives from teachers, students with disabilities, and families, the framework has not been validated, and potential bias in the selection of literature.

 19. Strengthen future research. The next research direction should include empirical validation of the proposed framework and diagnostic tool, comparative studies across different national.

 I appreciate the authors’ efforts in addressing an important and timely issue, and I hope these comments will help strengthen the manuscript and further enhance its contribution to the field.

 Reviewer.

Is the work clearly and accurately presented and does it cite the current literature?

Partly

If applicable, is the statistical analysis and its interpretation appropriate?

Not applicable

Are all the source data underlying the results available to ensure full reproducibility?

Partly

Is the study design appropriate and is the work technically sound?

Partly

Are the conclusions drawn adequately supported by the results?

Partly

Are sufficient details of methods and analysis provided to allow replication by others?

No

Reviewer Expertise:

Character and Inclusive Education

I confirm that I have read this submission and believe that I have an appropriate level of expertise to confirm that it is of an acceptable scientific standard, however I have significant reservations, as outlined above.

# Response

**Published:** 2026-09-08

**Authors:** Pongmanut Deeod

Thank you for the detailed and constructive review. In the revision, we will clarify that the core contribution is the paradigm misalignment concept and moral trap proposition rather than the underlying theories themselves; make the document search strategy and coding process transparent; strengthen the evidentiary link between the four structural conditions and source documents; reframe the moral trap explicitly as an untested theoretical proposition; moderate absolute language throughout; and update references with 2020–2026 literature. We will also add a limitations section. A revised version will follow shortly.
